# The Predictive Value of Growth Differentiation Factor-15 in Recurrence of Atrial Fibrillation after Catheter Ablation

**DOI:** 10.1155/2020/8360936

**Published:** 2020-08-21

**Authors:** Ying Wei, Shuwang Liu, Haiyi Yu, Yuan Zhang, Wei Gao, Ming Cui, Lei Li

**Affiliations:** ^1^Department of Cardiology and Institute of Vascular Medicine, Peking University Third Hospital, China; ^2^Key Laboratory of Cardiovascular Molecular Biology and Regulatory Peptides, Ministry of Health, China; ^3^Key Laboratory of Molecular Cardiovascular Science, Ministry of Education, China; ^4^Beijing Key Laboratory of Cardiovascular Receptors Research, Beijing 100191, China

## Abstract

The mechanisms underlying the recurrence of atrial fibrillation (AF) after radiofrequency catheter ablation (RFCA) are not well concerned. The study sought to explore the association between growth differentiation factor-15 (GDF-15) and the incidence of recurrent events among AF patients after the ablation procedure. We prospectively included 150 consecutive AF patients who underwent RFCA. Clinical information about the patients was collected. Blood samples on the second morning of hospital admission and three months after RFCA were collected, and enzyme-linked immunosorbent assay (ELISA) was used to measure the concentration of GDF-15. All participants were followed up at specific times (1st/3rd/6th/12th/18th/24th months) after RFCA to record recurrences events. During a median follow-up of 14.0 months, AF recurrence occurred in 37(24.7%) patients. Baseline serum GDF-15 level in the persistent AF group was significantly higher than the paroxysmal AF group [1140(854~1701)ng/L vs. 1062(651~1374)ng/L, *P* = 0.039]. Baseline serum GDF-15 level in the recurrence group was significantly higher than the nonrecurrence group [1287(889~1768) ng/L vs. 1062(694~1373)ng/L, *P* = 0.022]. Serum GDF-15 level at three months after RFCA was significantly lower than the baseline [870 (579~1270) ng/L vs. 1155 (735~1632)ng/L, *P* < 0.001]. The baseline GDF-15 correlated significantly with LAP (*r* = 0.296, *P* < 0.001) and LAAV(*r* = −0.235, *P* = 0.003). Kaplan-Meier analysis showed a significantly lower event-free survival time in the high baseline GDF-15 (≥1287.3 ng/L) group than the low baseline GDF-15 (<1287.3 ng/L) group (17.1 months vs. 20.4 months, Log Rank *P* = 0.017). In the multivariate Cox regression, baseline GDF-15(HR 1.053, 95% CI 1.007-1.100, *P* = 0.022) and LAD (HR 1.124, 95% CI 1.011-1.250, *P* = 0.030) were independent predictors of AF recurrence after RFCA. Our study indicated increased preprocedural GDF-15 is associated with left atrial remodeling and acts as a predictor of AF recurrence after ablation.

## 1. Introduction

As the most common sustained arrhythmia, atrial fibrillation (AF) represents an evolving, global epidemic problem and a major public health challenge worldwide [1]. With the aging of population intensifies, the morbidity of AF is increasing. The prevalence of AF in the developed world is 1% to 2% in the general population [2, 3] and increases to 8-15% at 80 years of age or older [4, 5]. AF remains one of the major causes of stroke, heart failure, sudden death, and cardiovascular morbidity in the world. The increasing prevalence of AF bears a heavy public health burden.

In the past decade, although advanced ablation techniques dramatically improved the outcomes [2, 6, 7], AF recurrence is still common and a significant number of patients continue to suffer complications [8], such as cardiac tamponade, pulmonary vein (PV) stenosis, and vascular injuries. It is reported that the recurrence rates after ablation were 20~50% [9]. The efficacy of ablation is considered quite challenging due to many factors, including the type of AF, ablative techniques, operator experience, clinical parameters, and strategy of follow-up [10]. However, a reliable parameter for predicting recurrent events of AF after ablation is absent.

GDF-15 was described as a divergent member of the human transforming growth factor *β* (TGF-*β*) superfamily. Its expression would increase in response to diverse cellular stress signals, such as inflammation, hypoxia/anoxia, acute tissue injuries, and the tumor process [11]. Cardiovascular disease is also a major driver of GDF-15 production [12]. Our group has demonstrated in vivo that GDF-15 could enhance the proliferation of fibroblasts and may participate in the progression of myocardial fibrosis [13]. Research in the past showed that GDF-15 played an active role in left ventricular remodeling [14]. Moreover, GDF-15 in serum was higher in patients with atrial fibrillation [15–17], and it could be used as a prognostic indicator for bleeding and death [18]. It is recognized that human transforming growth factor *β*_1_ (TGF-*β*_1_), which is also a member of the TGF-*β* family, exhibited a profibrotic effect on cardiomyocytes and thus increased the chances to develop AF [19]. The animal model showed that it could be used as a therapy target against fibrosis [20]. Since in AF patients, levels of TGF-*β*_1_ and GDF-15 in serum and atrial tissue both elevate [15–17, 21, 22], and they are both members of the TGF-*β* family, we infer that GDF-15 also have an important role in atrial fibrosis. However, few studies had focused on how GDF-15 correlates with atrial structural remodeling and AF types in AF patients. And there has been no research concerning the relationship between GDF-15 and AF recurrences up until now. In this study, we planned to explore whether the serum GDF-15 level measured preprocedure could predict AF recurrent events in AF patients after RFCA.

## 2. Methods

### 2.1. Patient Enrollment

Our study was a prospective, single-center study. From October 2017 to October 2019, a total of 150 consecutive AF patients admitted to Peking University Third Hospital for their initial circumferential pulmonary vein isolation (CPVI) based catheter ablation were qualified and recruited according to our criteria.

The exclusion criteria included acute coronary syndromes, congenital heart disease, valvular heart disease of any degree, severe heart failure (NYHA≥III class), severe liver disorders [aspartate  aminotransferase  (AST) > 40 U/L or alanine  aminotransferase  (ALT) > 40 U/L] or kidney disorders(creatinine > 4.0 mg/dl), current infection, hematological disease, and thyroid-related hospital diagnoses. The study was approved by the Ethics Review Boards of Peking University Third Hospital (Approval number: 077-02, Beijing, China). Written informed consent was obtained from all participants.

### 2.2. Clinical Data and Measurement

#### 2.2.1. Collection of Clinical Information

The demographic and clinical information of all patients were collected, including age, gender, body mass index (BMI), heart rate, previous history, and the use of medications. CHA_2_DS_2_-VASc and HAS-BLED scores for stroke and bleeding risk were also calculated.

#### 2.2.2. Measurement of Blood Parameters

Complete blood count, fasting blood glucose (FBG), hemoglobin A1c (HBA1c), creatinine (Cr), high-sensitivity C-reaction protein (hs-CRP), N-Terminal pro-brain natriuretic peptide (NT-proBNP), blood urea nitrogen (BUN), and uric acid (UA) were measured by clinical laboratories in Peking University Third Hospital using the standard laboratory procedures. The estimated glomerular filtration rate (eGFR) was obtained by the Simplified MDRD formula: GFR (ml/min · 1.73m2) = 186 × [Scr(mg/dl)] − 1.154 × age (years) − 0.203 [×0.742 for female, ×1.233 for Chinese].

#### 2.2.3. Measurement of GDF-15

Venous blood samples were obtained following overnight fasting (8 hours), after a 30-minute rest in the sitting position. Samples were taken from the cubital vein into blood tubes and immediately stored on ice at 4°C. Serum samples were processed within 30 minutes after collection by centrifugation at 3000 g for 15 minutes at 4°C. To avoid repeated freeze-and-thaw cycles, each serum sample was divided into 0.2 mL aliquots and frozen immediately at -80°C. Serum GDF-15 level was determined using a commercially available sandwich ELISA kit (R&D Company, USA). The samples were analyzed using the instructed procedures.

#### 2.2.4. Echocardiography

Transesophageal echocardiography (TEE) using GE Vivid E9 equipped with a multiplane TEE probe was performed within 24 hours before RFCA. Left atrial appendage images were obtained both in the basal short-axis view with a transverse scan and in the left ventricle-left atrial 2-chamber view with a vertical scan [23]. The flow velocity in and out of left atrial appendage was obtained by pulsed-wave Doppler interrogation at the left atrial appendage ostium and was measured as the average value of 10 consecutive fibrillatory emptying waves [24]. All TEE images were stored for offline analysis (QLAB cardiac 3DQ, Philips Medical Systems).

Two-dimensional transthoracic echocardiography was performed before the TEE study, using GE Vivid E9 and a 3.5 MHz transducer. Standard views including M-mode, 2D images, and Doppler and color-Doppler data were acquired from the parasternal and apical views (4-, 2-, and 3-chamber view), and parameters were recorded [24, 25].

### 2.3. Electrophysiological Study

Multidetector computed tomography (MDCT) scanning of the left atrial (LA) and pulmonary veins was performed to guide the ablation procedure by a 64-slice scanner (SOMATOM Definition Flash, Siemens, Munich, Germany).

All participants under intravenous sedation underwent the CPVI based ablation procedure guided by 3-D mapping in the CARTO 3 system [7, 26]. The technique of CPVI was described briefly as follows. Firstly, two transseptal punctures were performed to explore LA, followed by 5,000 IU of unfractionated heparin administration. The geometry of LA and PV trunk was reconstructed with a PentaRay Catheter (Biosense Webster, USA) in the CARTO 3 system compared with MDCT.

An irrigated radiofrequency ablation was performed along each PV antrum in a point-by-point fashion for encircling PVs. Smart Touch force-sensing catheter (Biosense Webster, USA) was used during the procedure. Radiofrequency energy was applied at 25~40 W with a temperature of 43°C and an irrigation flow of 8~25 mL/min until a bipolar voltage of <0.1 mV was achieved, with a maximum of 60 seconds per point. Endpoints of the procedure were completeness of PV isolation from LA. Successful PV isolation was defined as the elimination (or dissociation) of all the PV potentials recorded from a PentaRay Catheter. Ablation of LA roofline and other nonpulmonary vein foci triggering AF were added according to electrophysiological mapping results. All block lines were confirmed by the electrophysiologic study. If AF continued after the ablation, biphasic direct current shocks with the energy of 100~150 J were administered to restore sinus rhythm, or ibutilide was given intravenously. Heparin was repeated during the procedure. Activated clotting time (ACT) of whole blood, the target value of which was 200~350 s, was tested regularly to adjust the dosage of heparin every 30~60 minutes.

### 2.4. Follow-Up and AF Recurrence Assessment

Amiodarone was administered to the patients without contraindications for 3 months after the procedure. If there was no atrial tachyarrhythmia, an attempt was made in all patients to cease amiodarone and any other antiarrhythmic drugs (AADs). Vitamin K antagonist or dabigatran etexilate or rivaroxaban were prescribed for a minimum of 3 months without contraindications and potentially discontinued in the cases of a low thromboembolic score (CHA_2_DS_2_-VASc score 0 or 1).

All participants had regular follow-up visits with clinical evaluation. They received face-to-face interviews at the outpatient clinic every 3 months in the first year, and every 6 months afterwards. Blood samples of the patients were collected three months after catheter ablation. The total follow-up period was 2 years. 12-lead ECG and 72-hour Holter were performed at 1st, 3rd, 6th, 12th, 18th, and 24th months after the procedure. Every symptomatic patient was referred to a new ECG. The endpoint of the study is recurrent events. Recurrence of AF postablation is defined as a recurrence of AF/atrial flutter/atrial tachyarrhythmia of at least 30 s duration that is documented by an ECG or device recording system after a blanking period of 3 months [9]. Two electrophysiologists performed the clinical follow-up and data collection independently.

### 2.5. Statistical Analysis

The Kolmogorov-Smirnov test was used to test normality and a *P* value > 0.05 was defined as normally distributed data. Data were expressed as the mean value ± standard deviation (*X* ± SD) in continuous variables of a normal distribution, median ± quartile ranges (QR) in abnormal distribution and percentage, or proportion in categorical variables as appropriate. Continuous variables that showed normal distribution were compared using the Student's *t*-test, whereas the Mann-Whitney *U* test was used for nonnormally distributed samples, and chi-square tests for categorical data. Wilcoxon matched-pairs signed-rank test was used to compare the GDF-15 levels at baseline and three months after RFCA. Receiver operating characteristic (ROC) analysis was made to determine the cut-off value of GDF-15 to predict AF recurrence. A Kaplan-Meier curve censored for the recurrent event with a log-rank test served for the cumulative recurrence rate after ablation. Estimations of recurrence risks were performed using the Cox proportional hazard models by univariate and multivariate analysis. Spearman's correlations were used to examine the relationship between GDF-15 and other variables. Results were expressed as the *P* value and hazard ratio (HR) in confidence interval (CI) of 95%.

All statistical analyses were computed in a commercially available statistical calculation program (SPSS 23.0, SPSS Inc., Chicago, Illinois). Figures were analyzed using GraphPad Prism (GraphPad Software Inc., San Diego, California). Statistical significance was defined as a two-sided *P* value < 0.05 for all comparisons.

## 3. Results

### 3.1. Baseline Characteristics

A total of 150 patients undergoing their initial ablation [85 men and 65 women, mean age (64 ± 11) years old] were analyzed.

After a median follow-up time of 14.0 months, 37 (24.7%) patients experienced recurrent events confirmed by atrial tachyarrhythmia or medical records, and 113 patients (75.3%) had maintained stable sinus rhythm.

Patients were divided into 2 groups according to whether AF recurred. Baseline characteristics are summarized in Table 1. It was found that the recurrence group was older, had higher rates of persistent AF, higher CHA_2_DS_2_-VASc and HAS-BLED scores and a lower proportion of NOAC usage (*P* < 0.05). As for laboratory results, AF recurrence group had a significantly higher level of NT-proBNP, and a significantly lower level of eGFR (*P* < 0.05). Regarding echocardiographic data, LAD, LAA, LAP, and RAA were significantly higher in the recurrence group. LAAV and LVEF were significantly lower in the recurrence group. Regarding electrophysiological study data, the recurrence group has a higher percentage of CPVI-only patients. Other baseline characteristics were similar between the 2 groups (*P* > 0.05).

### 3.2. Serum GDF-15 Level in AF Patients

Baseline serum GDF-15 level in the persistent AF group was significantly higher than the paroxysmal AF group [1140(854~1701)ng/L vs. 1062(651~1374)ng/L, *P* = 0.039]. Baseline serum GDF-15 level in the recurrence group was significantly higher than the non-recurrence group [1287(889~1768)ng/L vs. 1062(694~1373)ng/L, *P* = 0.022]. Blood samples of 95 (63.3%) patients were collected three months after RFCA. Serum GDF-15 level at three months after RFCA was significantly lower than the baseline [870 (579~1270) ng/L vs. 1155 (735~1632)ng/L, *P* < 0.001].

### 3.3. Predictive Value of Baseline GDF-15 for AF Recurrence

**Figure 1:**
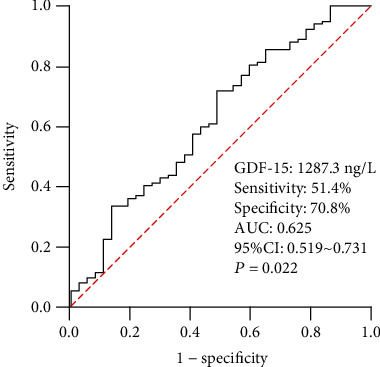
ROC curve analysis to determine the predictive value of GDF-15 for AF recurrence. AF: atrial fibrillation; AUC: area under the curve; CI: confidence interval; ROC: receiver operating characteristic; GDF-15: growth differentiation factor-15.

**Figure 2:**
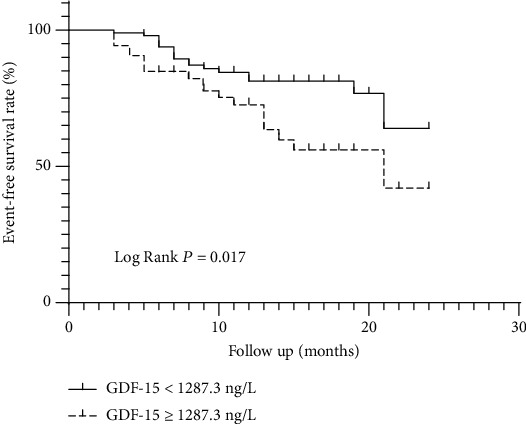
Kaplan-Meier curves of event-free survival rate by GDF-15.

The cut-off value of GDF-15 obtained by ROC curve analysis was 1287.3 ng/L for prediction of AF recurrence (sensitivity: 51.4%, specificity: 70.8%). The area under the curve (AUC) was 0.625 (95% CI 0.519-0.731, *P* = 0.022, Figure 1). All 150 AF patients were divided into two subgroups based on the baseline GDF-15 cut-off value (1287.3 ng/L). Kaplan-Meier analysis (Log Rank method) showed a significantly lower event-free survival time in the high GDF-15 group than the low GDF-15 group (17.1 months vs. 20.4 months, Log Rank *P* = 0.017, Figure 2).

### 3.4. Univariate and Multivariate Analyses of AF Recurrence after RFCA

Univariate and multivariate analysis results for AF recurrence are shown in Table 2. The variables significantly associated with AF recurrence included age, persistent AF, diabetes mellitus, NT-proBNP, LAD, LAAV, ablative strategy (CPVI-only), and baseline GDF-15. In the multivariate Cox regression, forward stepwise analysis adjusted by age, persistent AF, diabetes mellitus, NT-proBNP, eGFR, LAD, LAAV, ablative strategy (CPVI-only), and baseline GDF-15 indicated that baseline GDF-15 (HR 1.053, 95% CI 1.007-1.100, *P* = 0.022) and LAD (HR 1.124, 95% CI 1.011-1.250, *P* = 0.030) were both independent predictors of AF recurrence after RFCA.

### 3.5. The Correlation between Baseline GDF-15 and Other Characteristics

Among the clinical characters, laboratory results, and echocardiographic data, Spearman rank correlation analysis showed that the baseline serum GDF-15 level was positively correlated with age (*r* = 0.485, *P* < 0.001), CHA_2_DS_2_-VASc score (*r* = 0.349, *P* < 0.001), HASBLED score (*r* = 0.295, *P* < 0.001), NT-proBNP (*r* = 0.232, *P* = 0.005), LAP (*r* = 0.296, *P* < 0.001), and also negatively correlated with eGFR (*r* = −0.332, *P* < 0.001), LAAV (*r* = −0.235, *P* = 0.003) (Table 3). However, there was no correlation between GDF-15 and other variables (*P* < 0.05).

## 4. Discussion

This prospective study investigated the association between preprocedural serum GDF-15 level and recurrent events in 150 patients with AF after their initial catheter ablation. Our results showed that patients with higher baseline GDF-15 level were at greater risk and prone to suffer recurrent events. To the best of our knowledge, this is the first study investigating the role of GDF-15 to predict AF recurrence after catheter ablation.

Common mechanisms of atrial fibrillation include reentry, pulmonary vein trigger, abnormal autonomic nerve modulation, inflammation, and atrial remodeling. There are still recurrences of AF in some patients after catheter ablation, which mainly targets correcting atrial electrical remodeling. This indicates that correcting atrial electrical remodeling is not the only therapy target for AF. It has become popular to study the role and mechanism of atrial structural remodeling in the occurrence and development of AF. Atrial fibrosis is a key element of atrial structural remodeling. Fibrosis causes an increase in the volume of the dysfunctional extracellular matrix and is associated with cellular alterations such as hypertrophy, apoptosis, and membrane dysfunction within the atrial myocardium; In turn, these cause pathological alterations to atrial conduction, such as increased anisotropy, conduction block, and reentry, which can lead to AF [27]. LA enlargement and dysfunction are common in patients with AF and are always used to evaluate the severity degree of atrial structural remodeling [28]. Previous studies have shown that patients with larger LAD tend to have more chances to develop AF [29–32]. Also, AF patients with larger LAD or smaller LAAV, LVEF are at a greater risk of suffering from recurrence events [33–35]. Our study has also found that LAD and LAP were significantly higher in the patients with AF recurrence, and LAAV was significantly lower in the patients with AF recurrence. LAD was an independent predictor of AF recurrence after catheter ablation in the Cox model. Studies revealed that LAD, LAP, and LAAV are all important indicators of LA remodeling. In conclusion, our study suggested that the AF recurrence group had a severer degree of atrial structural remodeling.

We further found that in the persistent AF group and recurrence group, the serum GDF-15 level also increased accordingly. Cox model suggested that GDF-15 was an independent predictor of AF recurrence after RFCA. GDF-15 was a member of the human transforming growth factor *β* (TGF-*β*) superfamily. It was identified to have protective effects on inhibiting the proliferation and differentiation of cells, reducing ischemia-reperfusion injury and antiapoptosis [36, 37]. Our previous study showed that GDF-15 could enhance the proliferation of fibroblasts and may participate in the progression of myocardial fibrosis [13], and GDF-15 blocks norepinephrine-induced myocardial hypertrophy via a novel pathway involving inhibition of epidermal growth factor receptor transactivation [38]. Atrial structural remodeling is closely related to atrial fibrosis and extracellular matrix. Matrix metalloproteinases (MMPs) and their tissue inhibitors (TIMPs) are essential for the cardiac extracellular matrix (ECM) remodeling. Nair and Gongora [39] reported that the plasma GDF-15 level was positively correlated with MMP, TIMPs. Zhou et al. [40] showed that the GDF-15 level in plasma and the GDF-15 mRNA level in atrial tissue of AF were both higher than those of participants with sinus rhythm, and the expression of GDF-15 was found to be positively related to the degree of cardiac fibrosis. Also, there exist longstanding electric stimulation, oxidative stress, and inflammatory response in the atrium of persistent AF patients. All these reactions could aggravate atrial structural remodeling and may result in an increased serum level of GDF-15. The above results indicated that GDF-15 might play an important role in atrial structural remodeling by collagen synthesis and transformation, thus participating in AF recurrence and the evolvement from paroxysmal AF to persistent AF. More importantly, we further found that the expression of GDF-15 level decreases after RFCA. As catheter ablation is one of the most effective ways to stop AF by PV isolation and correcting atrial electrical remodeling, AF burden decreases after RFCA, which could slow down the progression of atrial fibrosis. We infer this may have some association with a decreased level of GDF-15 after RFCA.

Our study has also found that the baseline serum GDF-15 level was positively correlated with LAP and negatively correlated with LAAV. After AF recurrence, the left atrium has changes in tissue, cells, electrical remodeling and hemodynamics, resulting in LAP progressively heightened. The elevation of LAP also shortens the effective refractory period of cardiomyocytes and increases the chances of developing AF [41]. When left ventricular diastolic dysfunction happens, afterload of left atrium increases, then LAP heightens, causing the expansion of left atrium and myocardial fibrosis [42–44]. It is concluded that LAP is also an indicator of atrial structural remodeling. The left atrial appendage is adjacent to the LA, and they have many anatomic and histologic similarities. As a surrogate factor of LA function during AF, lower LAAV represents severe atrial remodeling and induces left atrial appendage thrombus. The clinical trial had indicated that AF patients who underwent ablation with lower preprocedural LAAV were at high risk of recurrence [34]. The positive correlation of GDF-15 and LAP and negative correlation of GDF-15 and LAAV indicated that GDF-15 and atrial structural remodeling are closely related. The higher serum GDF-15 level, the severer atrial structural remodeling, increasing the chances to AF recurrence. Therefore, we infer that GDF-15 might promote collagen synthesis and transformation to take part in atrial structural remodeling, making an effect on AF recurrence. We firstly found that GDF-15 acts as a predictor in recurrence of AF after ablation, which not only opens new research perspectives for revealing the mechanism of AF but also provides potential therapeutic targets that reduce AF recurrences after catheter ablation. GDF-15 might become a new biomarker of AF in the future.

The expression of GDF-15 would increase in response to inflammation and it acts as an inflammatory biomarker. However, our study found no association between other inflammatory markers (e.g. WBC and hs-CRP) with GDF-15. Our sample size is relatively small, and the expression of GDF-15 would differ in the condition of different populations and disease states. Although WBC and hs-CRP are both acute phase proinflammatory biomarkers, they could only reflect in part the severity of inflammation. Meanwhile, the relationship between WBC, hs-CRP with GDF-15 has not been reported before. We need to enroll more participants to further explore the association.

## 5. Limitations

This was a single-center study with its inherent limitations. Firstly, the sample size was relatively small which may cause a statistical bias. Our results need to be confirmed in future large multicenter prospective trials. Secondly, for paroxysmal AF patients, the rhythm may be different at the time of blood collection, which could make an effect on GDF-15 expression. Thirdly, the relationship between GDF-15 and echocardiographic data could not definitely prove that GDF-15 participates in the atrial structural remodeling process, which needs to be tested by some animal models and cell molecular biological experiments. Therefore, future studies are required to further evaluate the potential mechanism of GDF-15 in AF patients after catheter ablation.

## 6. Conclusions

Firstly, we found that serum GDF-15 level had positive correlations with LAP and negative correlations with LAAV, which suggested that GDF-15 may have clinical correlations with the atrial structural remodeling in AF patients. Moreover, our study found that the expression of GDF-15 in serum elevated in the persistent AF group compared with the paroxysmal AF group, also elevated in the recurrence group compared with the non-recurrence group, and decreased after RFCA. GDF-15 was an independent predictor for AF recurrence. This suggests that GDF-15 might be involved in the development of AF and become a new biomarker to predict AF recurrence after catheter ablation.

## Figures and Tables

**Table 1:** Baseline characteristics of patients with and without AF recurrence.

Characteristics	Recurrence (*n* = 37)	No recurrence (*n* = 113)	*P* value
Clinical characters			
Age (years)	69 ± 9	63 ± 11	0.005^∗^
Male, *n* (%)	18 (48.6)	67 (59.3)	0.257
BMI (kg/m^2^)	26 ± 4	26 ± 4	0.489
Heart rate (bpm)	80 ± 16	82 ± 16	0.457
AF duration (months)	24 (5~84)	13 (3~57)	0.152
Persistent AF, *n* (%)	23 (62.2)	39 (34.5)	0.003^∗^
Stroke, *n* (%)	15 (40.5)	30 (26.5)	0.107
Hypertension, *n* (%)	26 (70.3)	68 (60.2)	0.271
Hyperlipemia, *n* (%)	15 (40.5)	39 (34.5)	0.507
Diabetes mellitus, *n* (%)	13 (35.1)	22 (19.5)	0.051
CHD, *n* (%)	8 (21.6)	11 (9.7)	0.059
Smoker, *n* (%)	6 (16.2)	13 (11.5)	0.643
CHF, *n* (%)	5 (13.5)	5 (4.4)	0.123
CHA_2_DS_2_-VASc score	3 (2~4.5)	2 (1~3)	0.004^∗^
HASBLED score	1 (1~2)	1 (0~1)	0.016^∗^
Medications			
NOAC, *n* (%)	26 (70.3)	107 (94.7)	<0.001^∗^
Amiodarone, *n* (%)	26 (70.3)	91 (80.5)	0.191
*β*-Blockers, *n* (%)	15 (40.5)	51 (45.1)	0.625
ACEI/ARB, *n* (%)	15 (40.5)	46 (40.7)	0.986
CCB, *n* (%)	17 (45.9)	35 (31.0)	0.097
Laboratory results			
WBC (×10^9^/L)	6.2 ± 1.6	6.0 ± 1.6	0.414
FBG (mmol/L)	5.7 (5.1~6.4)	5.3 (4.8~6.0)	0.075
HbAlc (%)	6.1 (5.8~6.6)	6.0 (5.7~6.4)	0.341
Cr (mg/dl)	0.90 ± 0.16	0.90 ± 0.17	0.866
hs-CRP (mg/L)	1.09 (0.44~3.09)	0.96 (0.38~2.94)	0.514
NT-proBNP (pg/ml)	885.0 (228.3~1628.0)	248.0 (94.3~604.9)	<0.001^∗^
BUN (mmol/L)	5.7 ± 1.4	5.7 ± 1.7	0.829
eGFR (ml/min)	74 ± 11	81 ± 13	0.010^∗^
UA (umol/L)	345 (295~448)	346 (286~402)	0.707
Echocardiographic data			
LAD (mm)	41 ± 4	38 ± 4	<0.001^∗^
LAA (cm^2^)	26 ± 7	22 ± 4	0.005^∗^
LVEDD (mm)	48 ± 4	47 ± 4	0.752
LAP (mmHg)	11 (9~14)	10 (9~11)	0.049^∗^
LVEF (%)	68 (64~71)	70 (67~73)	0.036^∗^
RAA (cm^2^)	20 (16~23)	16 (15~19)	<0.001^∗^
RVD (mm)	21.2 (20.0~23.7)	21.0 (19.2~22.5)	0.428
LAAV (m/s)	0.35 (0.28~0.46)	0.50 (0.30~0.65)	0.004^∗^
Electrophysiological study data			
Ablation time (s)	2298 (1846~2887)	2248 (1945~2971)	0.619
Heparin dosage (IU)	7000 (7000~8000)	7000 (7000~7000)	0.386
CPVI-only, *n* (%)	14 (37.8)	72 (63.7)	0.006^∗^

AF: atrial fibrillation; BMI: body mass index; CHD: coronary heart disease; CHF: chronic heart failure; NOAC: new oral anticoagulants; ACEI: angiotensin-converting enzyme inhibitors; ARB: angiotensin receptor blocker; CCB: calcium channel blocker; WBC: white blood cell; FBG: fasting blood glucose; HbAlc: hemoglobin A1c; Cr: creatinine; hs-CRP: high-sensitivity C-reaction protein; NT-proBNP: N-Terminal pro-brain natriuretic peptide; BUN: blood urea nitrogen; eGFR: estimated glomerular filtration rate; UA: uric acid; LAD: left atrial diameter; LAA: left atrial area; LVEDD: left ventricular end-diastolic dimension; LAP: left atrial pressure; LVEF: left ventricular ejection fraction; RAA: right atrial area; RVD: right ventricular diameter; LAAV: left atrial appendage flow velocity; CPVI: circumferential pulmonary vein isolation. ^∗^*P* < 0.05.

**Table 2:** Univariate and multivariate analysis for atrial fibrillation recurrence.

	Univariate analysis			Multivariate analysis		
	HR	95% CI	*P*	HR	95% CI	*P*
Age (years)	1.043	1.007-1.079	0.018^∗^	−	−	−
Sex (male)	1.296	0.679-2.474	0.432	−	−	−
BMI (kg/m^2^)	1.042	0.954-1.138	0.359	−	−	−
Persistent AF	2.280	1.172-4.435	0.015^∗^	−	−	−
Diabetes mellitus	1.980	1.006-3.899	0.048^∗^	−	−	−
hs-CRP (mg/L)	1.009	0.989-1.029	0.387	−	−	−
NT-proBNP (pg/ml)	1.001	1.000-1.001	<0.001^∗^	−	−	−
eGFR (ml/min)	0.979	0.957-1.001	0.057	−	−	−
LAD (mm)	1.169	1.081-1.265	<0.001^∗^	1.124	1.011-1.250	0.030^∗^
LVEF (%)	0.967	0.926-1.010	0.132	−	−	−
LAAV (m/s)	0.046	0.005-0.399	0.005^∗^	−	−	−
CPVI-only (%)	0.411	0.211-0.798	0.009^∗^	−	−	−
GDF-15 (×10^2^ng/L)	1.057	1.017-1.099	0.005^∗^	1.053	1.007-1.100	0.022^∗^

HR: hazard ratio; CI: confidence interval; GDF-15: growth differentiation factor-15; other abbreviations as in Table 1. ^∗^ : *P* < 0.05.

**Table 3:** Relationship between GDF-15 and Other Variables.

Variables	Correlation coefficient (*r*)	*P* value
Age (years)	0.485	<0.001^∗^
Sex (male)	0.057	0.488
BMI (kg/m^2^)	-0.045	0.591
Amiodarone	-0.095	0.250
AF duration (months)	-0.043	0.615
CHA_2_DS_2_-VASc score	0.349	<0.001^∗^
HASBLED score	0.295	<0.001^∗^
hs-CRP (mg/L)	0.139	0.103
NT-proBNP (pg/ml)	0.232	0.005^∗^
eGFR (ml/min)	-0.332	<0.001^∗^
LAD (mm)	0.084	0.314
LAA (cm^2^)	0.108	0.199
LVEDD (mm)	0.042	0.618
LAP (mmHg)	0.296	<0.001^∗^
LVEF (%)	-0.065	0.441
RAA (cm^2^)	0.094	0.262
RVD (mm)	0.001	0.992
LAAV (m/s)	-0.253	0.003^∗^

All abbreviations as in Table 1. ^∗^ : *P* < 0.05.

## Data Availability

The data used to support the findings of this study are available from the corresponding author upon request.
